# Characterization and pharmacokinetics of cinnamon and star anise compound essential oil pellets prepared via centrifugal granulation technology

**DOI:** 10.1186/s12917-024-04026-7

**Published:** 2024-05-09

**Authors:** Dandan Yi, Wei Xu, Lanqian Qin, Yifei Xiang, Yihao MO, Xia Liu, Yu Liu, Jianbo Peng, Zhengmin Liang, Jiakang He

**Affiliations:** 1 College of Animal Science and Technology, Guangxi University, 100 Daxue Road, Xixiangtang District, Nanning, 530004 Guangxi P. R. China; 2 Guangxi Key Laboratory of Animal Breeding, Disease Control and Prevention, Nanning, 530004 PR China; 3 Guangxi Zhuang Autonomous Region Engineering Research Center of Veterinary Biologics, Nanning, 530004 PR China; 4 Department of Animal Science and Technology, Guangxi Agricultural Vocational College, Nanning, 530007 PR China

**Keywords:** Compound essential oil pellets, Antibacterial, Enteric, Pharmacokinetics

## Abstract

Cinnamon and star anise essential oils are extracted from natural plants and provide a theoretical basis for the development and clinical application of compound essential oil pellets. However, cinnamon oil and star anise oil have the characteristics of a pungent taste, extreme volatility, poor palatability, and unstable physical and chemical properties, which limit their clinical use in veterinary medicine. In this study, the inhibitory effects of cinnamon oil and star anise oil on *Escherichia coli* and *Salmonella* were measured. Compound essential oil pellets were successfully prepared by centrifugal granulation technology. Subsequently, the in vitro dissolution of the pellets and their pharmacokinetics in pigs were investigated. The results showd that, cinnamon and star anise oils showed synergistic or additive inhibitiory effects on *Escherichia coli* and *Salmonella*. The oil pellets had enteric characteristics in vitro and high dissolution in vitro. The pharmacokinetic results showed that the pharmacokinetic parameters Cmax and AUC were directly correlated with the dosage and showed linear pharmacokinetic characteristics, which provided a theoretical basis for the development and clinical application of compound essential oil pellets.

**Supplementary Information:**

The online version contains supplementary material available at 10.1186/s12917-024-04026-7.

## Introduction

Bacterial diseases are one of the major causes of mortality worldwide. The abuse of antibiotics has allowed bacteria to develop extremely strong drug resistance, leading to both the production of superbacteria and the uncontrollable proliferation of bacterial disease [1]. In the aquaculture industry, diarrhoea, meningitis and cow mastitis caused by *Escherichia coli* [2], *Salmonella* [3, 4], *Streptococcus suis* [5], *Staphylococcus aureus* [6] and other bacteria are common, reducing animal production performance, causing enormous economic losses, polluting animal products and causing these products to become threats to human health. The incidence of multiantibiotic resistant bacterial infections continues to rise. Resistance to existing antibiotics has become a global health problem, leading to the urgent need for alternative antimicrobial strategies [7].

There is renewed interest in the development of antimicrobial agents from natural plant sources. Essential oils are plant-derived products that have been used for a long time in medicine, agriculture and food preservation due to their antibacterial activities. Essential oils are a promising substitutes for traditional antibiotics because they have extensive antibacterial activity and low toxicity to human symbiotic bacteria and can kill microorganisms without promoting drug resistance. Cinnamaldehyde and star anise oil are natural compounds that have received great attention in recent years [8]. Cinnamon and star anise oils have been proven to have certain antibacterial effects on *Salmonella*, *Escherichia coli*, *Staphylococcus aureus*, *Candida, Pseudomonas aeruginosa, Bacillus cereus* [9–16], etc. These botanical antimicrobial substances have great advantages and prospects in the development and application of antibacterial drugs. The existing drug formulations for cinnamon and star anise are mainly essential oils [17] and water or alcohol extracts [18, 19]. However, due to the high volatility, easy deactivation, strong smell, and unstable physical and chemical properties of these pharmacologically active substances, there are certain difficulties in their large-scale application [20, 21]. Changing drug formulations can provide new solutions for their development and utilization. The pellet formulation can allow drugs to be more evenly distributed in the gastrointestinal tract [22], and different types of pellets, such as slow and controlled release and enteric dissolution pellets, can be made. The enteric coating of these particles masked the strong taste of the drug, prevented it from being digested in gastric juice, reduced the irritation of the digestive tract by the drug, and released effective drugs after the pellets reach the intestine, achieved the goal of enteric coating [23]. .

Therefore, in this study, the combined bacteriostatic effect of cinnamon oil and star anise oil on *Salmonella* and *Escherichia coli in vitro* was explored. Then, we developed a type of pellet that included cinnamon oil, star anise oil and other excipients. The characteristics of the compound essential oil pellets were investigated in terms of their content, particle size distribution, angle of repose, fragility and stability, and the therapeutic effect of the drug was evaluated by an in vitro dissolution test. For the first time, gas chromatography–mass spectrometry/mass spectrometry (GC‒MS / MS) was used to simultaneously determine the contents of trans-anisole and cinnamaldehyde in pig plasma after the oral administration of compound essential oil pellets, and the pharmacokinetic parameters of these drugs were studied to provide a basis for later-stage clinical drug administration.

## Materials and methods

### Materials

Nutritional broth and Luria-Bertani (LB) agar were purchased from Beijing Landbridge Science and Technology Co., Ltd. (Beijing, China), Cinnamaldehyde standard (purity 98.00%) was obtained from Shanghai Yuanye Biological Technology Co., Ltd. (Shanghai, China), Trans-anethole standard (purity 98.00%) was obtained from Shanghai Yuanye Biological Technology Co., Ltd. (Shanghai, China), Cinnamon oil and star anise oil was obtained from Guangxi Bagui Tianxiang Co., Ltd. (Guangxi, China), Hydroxypropyl methylcellulose (HPMC), hydroxypropyl cellulose (HPC) and microcrystalline cellulose were purchased from MacKlin (Shanghai, China). Ethyl acetate was purchased from Sinopharm Chemical Reagent Co., Ltd. (Shanghai, China).

### Test strains

*E. coli*_*ATCC25922*_ was purchased from the China General Microbiological Culture Collection Center, and 19 strains of *E. coli* and 12 strains of *Salmonella* were isolated from clinical samples and provided by the Department of Pharmacology and Toxicology of China Agricultural University.

### Animals

Six healthy Landrace × Large White hybrid pigs were randomly divided into three groups for pharmacokinetic experiments and were purchased from JuCong Agriculture, Nanning, Guangxi. During the adaptation period, the temperature of the breeding environment was 18–25℃, the relative humidity was 50–65%, and commercial feed and water free of antibiotics and target drugs were freely available to the pigs housed individually in six pig pens. After the experiment, the pigs were released.

The study was conducted according to the recommendations of the Academy of Animal Research Guidelines and approved by the Animal Ethics Committee of Guangxi University (protocol number: GXU-2022-340).

All methods were carried out in accordance with relevant guidelines and regulations. All methods used for reporting the animal experiments are reported in accordance with ARRIVE guidelines (https://arriveguidelines.org) .

## Methods

### Bacteriostatic test of cinnamon oil and star anise oil

#### Determination of the minimum inhibitory concentration (MIC) of individual essential oils

The trial was conducted according to the methods recommended by The Clinical and Laboratory Standards Institute (CLSI), and the MICs of the essential oils was determined by microdilution. Cinnamon oil and star anise oil were added to a sterile 96-well plate with multiple dilutions from low to high concentrations from the first well to the tenth well, with 100 µL per well. 100 µL of bacterial solution was added to a concentration of 1 × 10^6^ CFU/mL. 200 µL of the bacterial solution was added to the 11th well as positive control, and 200 µL of broth was added to the 12th well as a negative control. The MIC test of quality control bacteria was carried out at the same time in each test. The 96-well plates were placed in a 37 ℃ incubator for 18 to 24 h, after which the results were observed and recorded. The experiment was repeated three times.

### Combined essential oil bacteriostatic test

The combined susceptibility test of the above two bacteria to cinnamon oil and star anise oil was carried out using the checkerboard method. The fractional inhibitory concentration (FIC) index is the graded bacteriostatic concentration index, which is one of the parameters of antimicrobial pharmacodynamics (PD) and indicates the combined susceptibility to two antibacterial drugs (when the two antibacterial drugs are used at the same time, there can be four cases of synergy, addition, unrelatedness and antagonism). The formula for calculating the FIC index is as follows:

$${\text{FITC=}}\frac{{{\text{Combined MIC of A}}}}{{{\text{Individual MIC of A}}}}{\text{ + }}\frac{{{\text{Combined MIC of B}}}}{{{\text{Individual MIC of B}}}}$$

An FIC index ≤ 0.5 indicates a synergy effect, 1 ≥ FIC index > 0.5 indicates an additive effect, 2 ≥ FIC index > 1 indicates unrelatedness, and an FIC index of > 2 indicates antagonism.

### Preparation of compound essential oil pellets

#### Preparation of the blank pellet core

First, 500 g of microcrystalline cellulose (MCC) was placed in a centrifugal granulator (model). The main engine speed was set to 200 r/min, the atomization pressure was 0.08 MPa, the blast flow was 10 L/min, and the temperature was 25 ∼ 30℃. Then, 5% HPMC was sprayed as a binder to complete the preparation process of the pellet cores. The pellet cores were removed and dried at 60 °C, and the blank pellet cores were set aside for future experimentation after reaching a size of 30–40 mesh.

### Preparation of compound essential oil pellets

First, 10 g of cinnamon oil and 5 g of star anise oil were evenly mixed and placed in a centrifugal granulator. The blank pellet core was used as the parent core. The binder was sprayed at a spraying speed of 12–17 r/min, and the jet pressure was set to 0.06 MPa. After the pellets reached 20–24 mesh, they were rounded for 5 min and removed for future use. Fifteen grams of HPMC was stirred for 1–2 h and passed through a 100 - mesh sieve, and compound essential oil pellets were obtained.

### Characterization of compound essential oil pellets

#### Particle size distribution

The particle size distribution of the pellets was determined by sieve analysis. The pellets were sieved with a standard sieve and weighed through a series of meshes, and the particle size distribution was plotted.

### Determination of the angle of repose

A funnel was fixed at a height of 3 cm above the graph paper. The materials were then passed through the funnel and formed a stacking cone. This process was performed until the top of the stacking cone just touched the bottom of the funnel. At this point, the diameter of the cone was measured, and the angle of repose was calculated as the tangent of the ratio of the height of the bottom of the funnel to the radius of the cone.

### Content determination

An appropriate amount of compound essential oil pellets was ground, placed in a 10 mL volumetric flask, added to an appropriate amount of ethyl acetate, and extracted by ultrasonication for 50 min. After allowing the solution to cool, ethyl acetate was added to dilute to the volumetric mark, and the resulting solution was filtered and measured by GC (7890 A, Agilent Technologies Co., Ltd., USA).

**In vitro release**.

The basket method was selected as the dissolution method and was performed according to the “Dissolution and Release Determination Method”, Method 2 (for enteric-coated preparations) of Appendix 162 of the “Chinese Veterinary Pharmacopoeia” 2020 Edition (Part I).

Release amount in acid: Approximately 900 mL of 0.1 mol/L hydrochloric acid solution was added to a dissolution cup and heated to maintain the solution temperature at 37 ± 0.5 °C, and 0.5 g of compound essential oil pellets was placed into a rotating basket. The machine was started at a speed of rotation of 100 rpm, and 1 mL aliquots were sampled at 0.5 h, 0.75 h, 1 h, 1.5 h, and 2 h and filtered through a 0.22 μm microporous water-phase filter. The time from sampling to filtration was 30 s. After sampling, ethyl acetate was added to the extract, and the resulting solution was brought to volume and analysed by GC.

Release amount in buffer: Approximately 900 mL of phosphate buffer (pH = 6.8) was heated to maintain the solution temperature at 37 ± 0.5 °C, and 0.5 g of compound essential oil pellets was placed into a rotating basket. The machine was started at a speed of 100 rpm, and 1 mL aliquots were sampled at 0.083 h, 0.166 h, 0.333 h, 0.75 h, 1 h, 1.5 h, and 2 h and filtered through a 0.22 μm microporous aqueous phase filter. The time from sampling to filtration was 30 s. After sampling, ethyl acetate was added to the extract, and the resulting solution was brought to volume and analysed by GC.

### Pharmacokinetics of compound essential oil pellets in pig model

After one week in the experimental setting, the pigs were labelled with numbers 1–6, randomly divided into 3 groups, and weighed before the experiment. The pigs were fasted for 12 h and given free access to drinking water. Compound essential oil pellets were orally administered at three different doses of 50, 100 and 200 mg/kg. Three cycles of crossover trials were performed with 6 pigs, with each cycle separated by 7 days. 5 mL of blood was collected from the anterior vena cava at different time points after administration, centrifuged at 3000 r/min for 10 min, after which the plasma was separated. The drug concentration in the plasma was detected by GC‒MS/MS after pretreatment.

The average drug-time curve of each group was drawn. WinNonlin version 5.2.1 was used to calculate the pharmacokinetic parameters.

### Determination of compound essential oil pellets in plasma samples

The compound essential oil pellet concentration was measured using GC‒MS/MS. The chromatographic conditions were as follows. A PEG-20 M column (30 m × 0.32 mm, 0.25 μm) was used. The temperature was increased from 90 ℃ to 160 ℃ at a heating rate of 10 ℃/min, held for 5 min, further increased from 160 ℃ to 280 ℃ at a heating rate of 20 ℃/min and held for 2 min. A matrix standard curve for determining the trans-anethole and cinnamaldehyde concentrations was plotted in the range of 0.02–4 µg/mL (R2 > 0.999). The mean recoveries of trans-anethole and cinnamaldehyde in plasma were 75.74-109.69% and 71.93-108.59%, respectively.

### Statistical analysis

Significant differences in the data in the study were analysed using SPSS Statistics 21 software (version 21, IBM). The data are presented as the means ± standard deviations (SDs).

## Results

### Bacteriostatic test results

3.1.1 MIC results of cinnamon oil and star anise oil alone or in combination against *E. coli* and *Salmonella*.

As shown in Table 1, the MICs of cinnamon oil and star anise oil for *E. coli* were 0.31–0.62 µg/mL and 50–100 µg/mL, respectively, the MICs of Cinnamon oil and Star anise oil for *Salmonella* were 0.19–0.39 µg/mL and 50–100 µg/mL, respectively. The inhibition of *E. coli* and *Salmonella* by the combined use of cinnamon oil and star anise oil was mainly synergistic or additive, and the MIC of cinnamon oil after the combined use of the two drugs was reduced to 1/4–1/2 that of the individual drug, and the MIC of star anise oil was reduced to 1/2 that of the individual drug. The FIC values of the combined use of cinnamon oil and star anise oil against 32 strains of *E. coli* and *Salmonella* ranged from 0.1 to 0.53, and the results are shown in Tables 1 and 2.

**Table 1:** MIC test results of the single and combined cinnamon oil and star anise oils against *E. coli* and *Salmonella*

Drugs Bacteria	Cinnamon oil		Star anise oil		FIC index	Combined antibacterial effect
	Individual MIC	Combined MIC	Individual MIC	Combined MIC		
***E. coli***	0.31–0.62	0.08–0.31	50–100	25–50	0.16–0.53	Synergistic/Additive
***Salmonella***	0.19–0.39	0.1–0.2	50–100	25–50	0.1–0.28	Synergistic

**Table 2:** Combined drug sensitivity test results of cinnamon oil and star anise oil

Bacteria	Synergistic	Additive	Unrelated	Antagonistic
***E. coli*** (20)	11 (20)	9 (20)	0 (0)	0 (0)
***Salmonella*** (12)	12 (12)	0 (0)	0 (0)	0 (0)
**total**	12 (32)	9 (32)	0 (0)	0 (0)

### Optimization of compound essential oil pellets

**Fig. 1:**
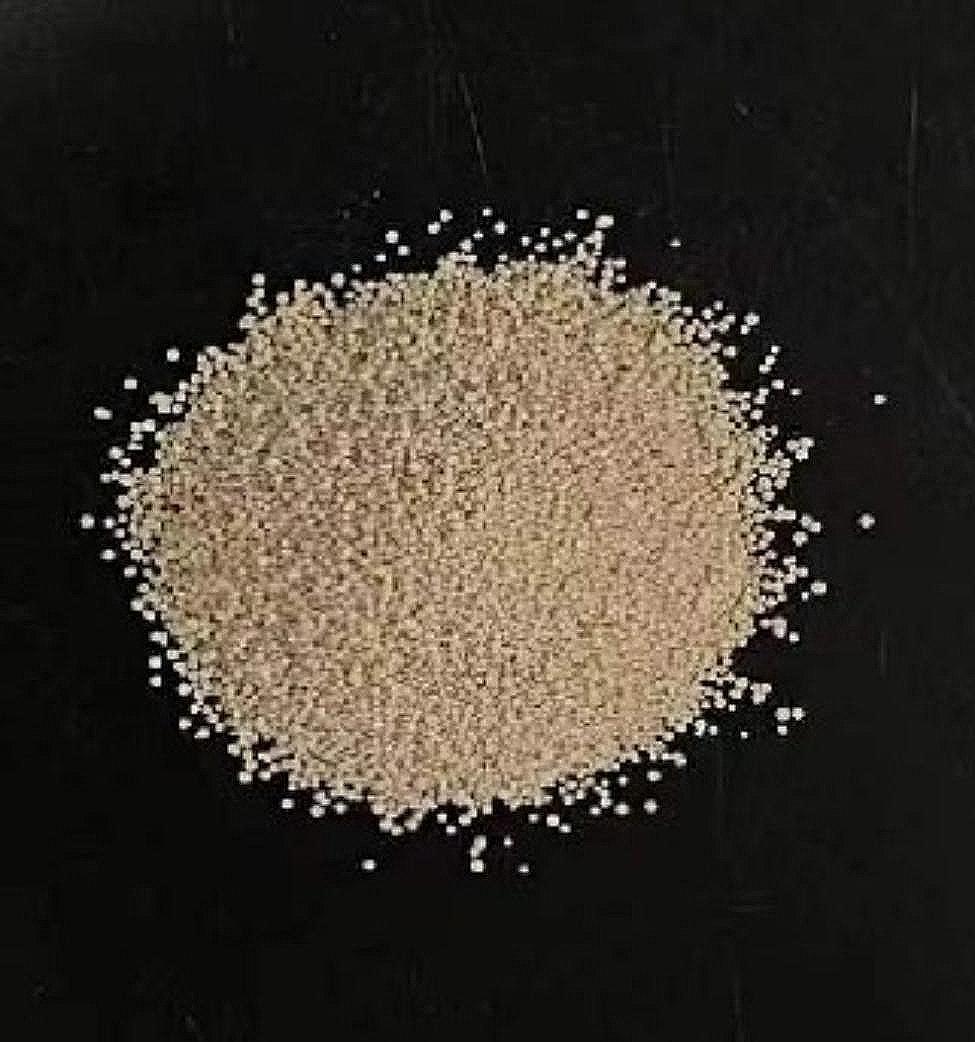
Prepared cinnamon oil and star anise oil compound essential oil pellets

The pellet cores for pellet preparation are mainly categorized as microcrystalline cellulose pellet cores, sucrose pellet cores and starch pellet cores. During the experiment, when the microcrystalline cellulose pellet core was selected, the compound essential oil pellets were successfully prepared, and the drug loading rate was high; when the sucrose and starch pellet cores were selected, the drug loading rate was low, and the drug did not easily adhere. Therefore, in this experiment, microcrystalline cellulose pellets were selected for pellet preparation. HPMC was selected as the binder, and the effects of different HPMC concentrations (1%, 3% and 5%) on the pellets were screened. When 1% and 3% HPMC were used as the adhesive, the viscosity was low, and the drug did not easily adhere. When 5% HPMC was selected, the viscosity was higher and the drug loss was less, so 5% HPMC was finally selected as the binder. The preparation results are shown in Fig. 1, the prepared compound essential oil pellets are light yellow spheres.

#### Characteristics of compound essential oil pellets

**Fig. 2:**
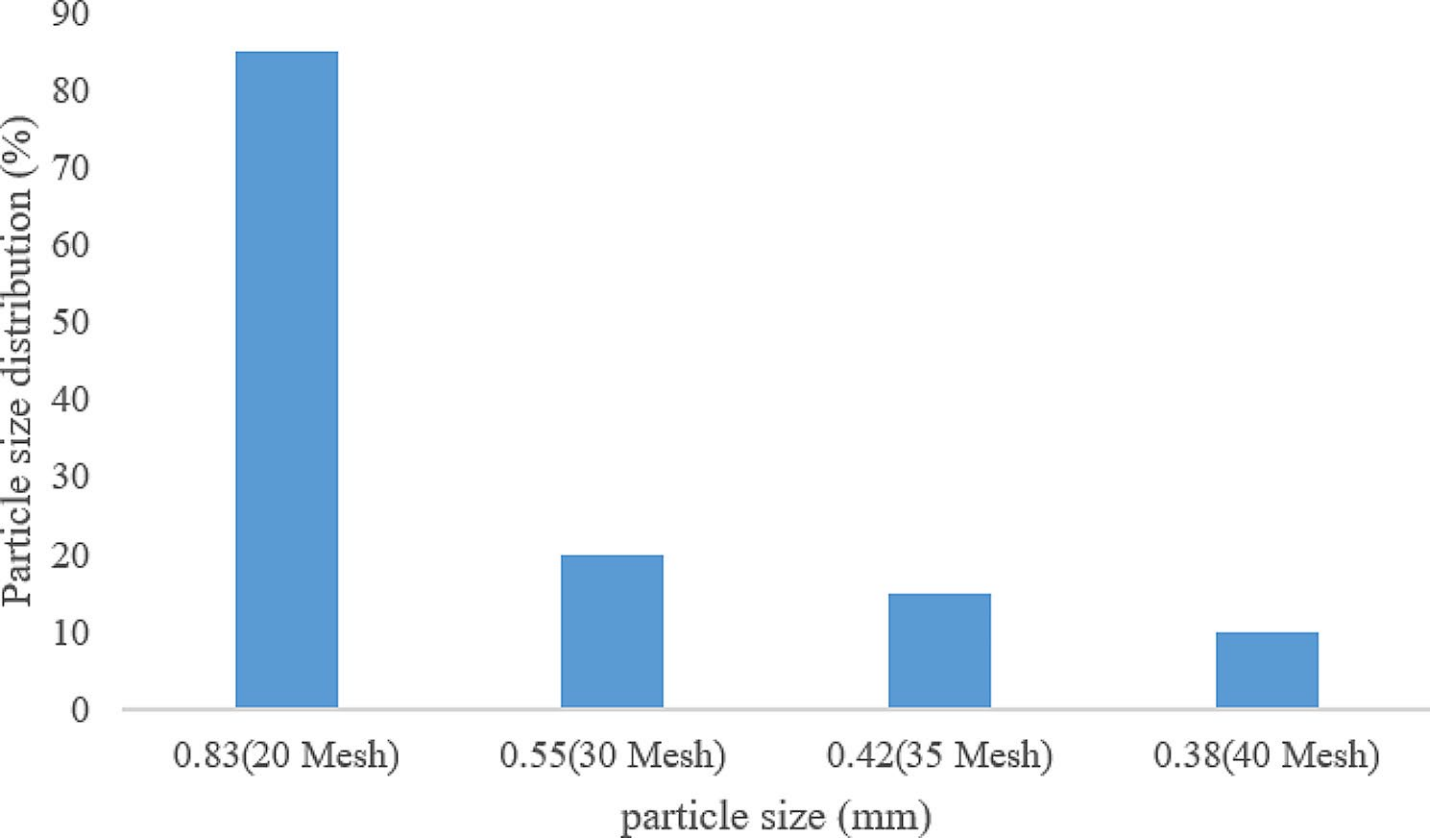
Particle size distribution

The particle size of the pellets was measured by the sieve analysis method to be 0.83 mm, and the particle size distribution diagram is shown in Fig. 2. The angle of repose of the pellets measured by the fixed funnel method under the optimal process conditions was 27.8°, which is less than 45°, indicating that the fluidity was good. Three batches of compound essential oil pellets were obtained, and the results are shown in Table 3. The stability of compound essential oil pellets was investigated through accelerated experiments, long-term experiments, and high-temperature and strong light experiments, the inspection results are shown in Tables 1, 2 and 3 of the supplementary materials. The results showed that there was no significant change in the drug content of the compound essential oil pellets under long-term and accelerated experimental conditions within 6 months. At the same time, under the conditions of high temperature and humidity, the drug content decreased with time, so the compound essential oil pellets should be stored in a cool and dark environment.

**Table 3:** Determination of trans-anethole and cinnamaldehyde content in three batches of compound essential oil pellets (*n* = 3)

Batch	Content (%)	
	Cinnamaldehyde	Trans-anethole
1	2.481 ± 0.133	1.291 ± 0.071
2	1.957 ± 0.147	1.169 ± 0.095
3	2.356 ± 0.135	1.315 ± 0.085

In vitro **release**.

**Fig. 3:**
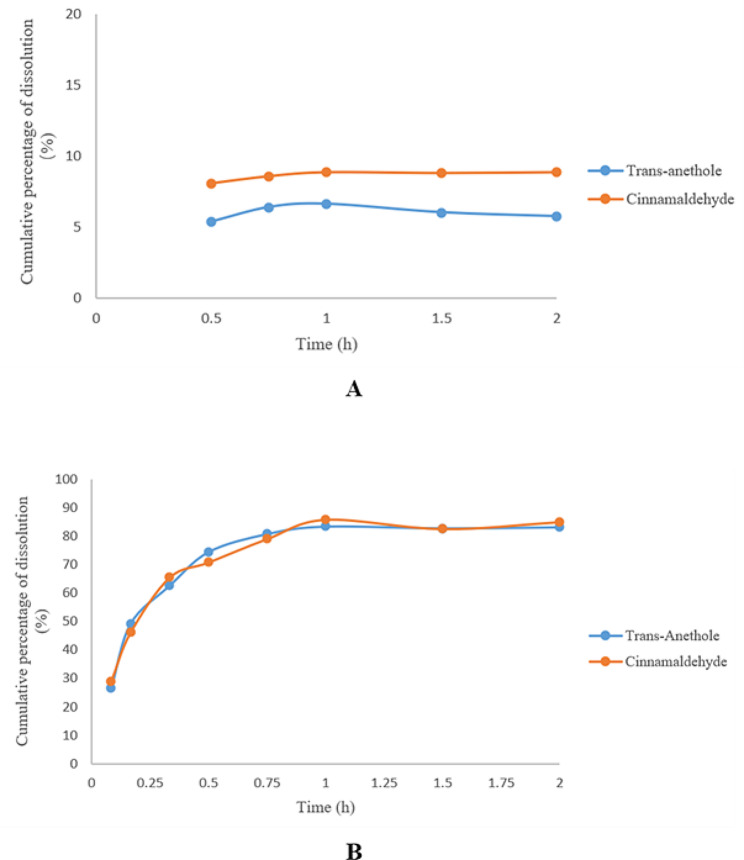
Release of compound essential oil pellets in acidic buffer(A)and buffer at pH 6.8 (B)

The dissolution results are shown in Fig. 3A and B. The dissolution rates of trans-anethole and cinnamaldehyde in the compound essential oil pellets in acidic buffer after 0.5 h were 5.34% and 8.05%, respectively. The dissolution rates of trans-anethole and cinnamaldehyde were 5.72% and 8.85%, respectively. At pH 6.8, the dissolution rates of trans-anethole and cinnamaldehyde were 26.55% and 28.92% at 0.083 h and 80.81% and 79.11% at 45 min, and the maximum dissolution rates of trans-anethole and cinnamaldehyde were 83.42% and 85.89%, respectively, after 1 h. The dissolution amount of compound essential oil pellets in acidic buffer solution after 2 h was less than 10%, and the dissolution amount in buffer solution at pH 6.8 after 0.75 h was more than 70%. Therefore, the compound essential oil pellets exhibited enteric properties.

### Pharmacokinetics of compound essential oil pellets in a pig model

**Fig. 4:**
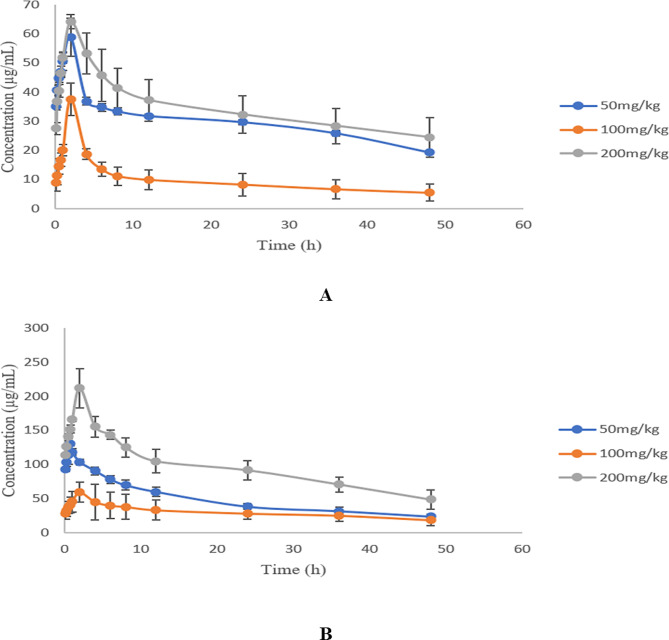
Time curves of cinnamaldehyde(A) and trans-anethole(B) in pig plasma after oral administration of three different doses of compound essential oil pellets

The drug-time curves of the plasma cinnamaldehyde and trans-anethole concentrations after oral administration of three different single doses of compound essential oil pellets (50 mg/kg, 100 mg/kg and 200 mg/kg) in a pig model are shown in Fig. 4A and B, respectively. It can be seen from the drug-time curve that the T_max_ value of cinnamic aldehyde in the compound essential oil pellets was the same in the three different dose ranges, and the C_max_ of the high dose group was greater than that of the other two dose groups, but the MRT_0−∞_ values were similar. The T_max_ values of trans-anethole in the compound essential oil pellets were different, but the overall difference was not significant. The C_max_ of the high dose group was greater than that of the other two dose groups, but the MRT_0−∞_ values were similar. A comparison of the pharmacokinetic parameters of the plasma cinnamaldehyde and trans-anethole concentrations after oral administration of three different doses of compound essential oil pellets (50 mg/kg, 100 mg/kg and 200 mg/kg) in a pig model is shown in Tables 4 and 5. The C_max_ values of cinnamaldehyde were 58.81 ± 6.16, 37.57 ± 5.56 and 64.18 ± 2.23 µg/L, respectively. The AUC_0−∞_ values were 1433.11 ± 21.30, 467.05 ± 141.28 and 1653.25 ± 295.40 h·µg/L; the MRT_0−∞_ values were 20.96 ± 0.21, 17.62 ± 1.84 and 20.68 ± 0.61 h; and the T_1/2_ values were 14.52 ± 0.14, 12.21 ± 1.27 and 14.33 ± 0.42 h, respectively. The pharmacokinetic parameter C_max_ values of trans-anisole in the compound essential oil pellets were 131.09 ± 3.94, 60.05 ± 18.73 and 211.71 ± 26.39 µg/L, respectively. The AUC_0−∞_ values were 2310.72 ± 220.38, 1433.55 ± 557.87 and 4567.26 ± 595.59 h·µg/L; the MRT_0−∞_ values were 17.55 ± 0.61, 20.44 ± 0.29 and 19.07 ± 0.43 h; and the T_1/2_ values were 12.16 ± 0.42, 14.16 ± 0.20 and 13.21 ± 0.29 h, respectively. At doses of 100 and 200 mg/kg, the pharmacokinetic parameters Cmax and AUC_0−∞_ of cinnamaldehyde and trans-anethole showed a certain linear relationship, while the T_max_, MRT_0−∞_, and T1/2 values were similar and independent of the dose. The results showed that the compound essential oil pellets exhibited linear pharmacokinetic characteristics in a pig model.

**Table 4:** Comparison of the pharmacokinetic parameters of plasma cinnamaldehyde after oral administration of three different single doses of compound essential oil pellets in a pig model (*n* = 6)

Parameter	Unit	50 mg/kg	100 mg/kg	200 mg/kg
T_max_	h	2.00	2.00	2.00
C_max_	µg/L	58.81 ± 6.16^a^	37.57 ± 5.56^d^	64.18 ± 2.33
AUC_0−∞_	h·µg/L	1433.11 ± 21.30^a^	467.05 ± 141.28^d^	1653.25 ± 295.40
Vz	L/kg	1205.24 ± 149.37^ab^	7671.46 ± 1809.86^d^	4593.67 ± 625.29
CL	L/h/kg	18.69 ± 1.81^ac^	142.48 ± 40.58^d^	56.85 ± 15.32
MRT_0−∞_	h	20.96 ± 0.21^a^	17.62 ± 1.84^d^	20.68 ± 0.61
T_1/2_	h	14.52 ± 0.14^a^	12.21 ± 1.27^d^	14.33 ± 0.42

^a^There was a statistically significant difference between the low-dose group and the medium-dose group (*p* < 0.01).

^b^There was a statistically significant difference between the low-dose group and the high-dose group (*p* < 0.01).

^c^There was a statistically significant difference between the low-dose group and the high-dose group (*p* < 0.05).

^d^There was a statistically significant difference between the medium-dose group and the high-dose group (*p* < 0.01).

**Table 5:** Comparison of the pharmacokinetic parameters of plasma trans-anethole concentration after oral administration of three different doses of compound essential oil pellets in a pig model (*n* = 6)

Parameter	Unit	50 mg/kg	100 mg/kg	200 mg/kg
T_max_	h	0.71 ± 0.10^AC^	2.33 ± 0.81	2.00
C_max_	µg/L	131.09 ± 3.94^AC^	60.50 ± 18.73^D^	211.71 ± 26.39
AUC_0−∞_	h·µg/L	2310.72 ± 220.38^BC^	1433.55 ± 557.87^D^	4567.26 ± 595.59
Vz	L/kg	665.12 ± 107.23^AC^	2674.85 ± 530.14^D^	1224.45 ± 63.61
CL	L/h/kg	15.06 ± 1.54^AC^	42.09 ± 12.59	31.67 ± 5.54
MRT_0−∞_	h	17.55 ± 0.61^AC^	20.44 ± 0.29^D^	19.07 ± 0.43
T_1/2_	h	12.16 ± 0.42^AC^	14.16 ± 0.20^D^	13.21 ± 0.29

^A^There was a statistically significant difference between the low-dose group and the medium-dose group (*p* < 0.01).

^B^There was a statistically significant difference between the low-dose group and the medium-dose group (*p* < 0.05).

^C^There was a statistically significant difference between the low-dose group and high-dose group (*p* < 0.01).

^D^There was a statistically significant difference between the medium-dose group and the high-dose group (*p* < 0.01).

## Discussion

Cinnamon oil and star anise oil have certain antibacterial effects on gram-negative bacteria and gram-positive bacteria. It has been reported that cinnamon oil has a significant antibacterial effect against *Staphylococcus aureus, Escherichia coli, Pseudomonas aeruginosa, Candida albicans* and *Aspergillus brasiliensis* with MICs of 156, 62.5, 125, 250 and 125 µg/mL [24], respectively. The MICs of star anise essential oil against *Aspergillus flavus* and *parasitic anise* is 0.5 and 1.0 µL/mL, respectively, and the MIC of trans-anisole against the *Staphylococcus aureus Newman strain* is 49.4 mg/mL [25]. In this study, the MICs of cinnamon oil and star anise oil against *E. coli* were 0.31–0.62 µg/mL and 50–100 µg/mL, respectively; the MICs of cinnamon oil and star anise oil against *Salmonella* were 0.19–0.39 µg/mL and 25–50 µg/mL, respectively. Compared with the results of a previous report, cinnamon oil showed a better antibacterial effect against *E. coli in vitro*. The combined use of drugs can reduce drug use, especially antibiotic use, and lessen the increase in bacterial drug resistance. The the cinnamon/clove oil combination has synergistic antibacterial activity against the foodborne bacteria *Staphylococcus aureus*, *Listeria monocytogenes, Salmonella typhimurium* and *Pseudomonas aeruginosa* [26]. The combination of black pepper and cinnamon can reduce *E. Fergusonii* growth and has bactericidal effects during storage [27]. In this study, the in vitro bacteriostasis tests of cinnamon and star anise oil showed that the two drugs had additive and/or synergistic inhibitory effects on *E. coli* and *Salmonella*, which provided a theoretical basis for the combined use of the two drugs.

Centrifugal granulation is considered a relatively advanced pellet preparation technology that has the characteristics of low production cost, flexible operation, flexible control parameters, and automatic operation [28]. When pellets are prepared by centrifugal granulation, the selection of the blank pellet core and binder is very important for the preparation process. Generally, blank pellets are mainly categorized as sucrose pellets, starch pellets or microcrystalline cellulose pellets. The selection of the blank pellet affects the physical form, drug loading and drug loss rate of the pellets; moreover, it has a certain influence on drug release, diffusion and penetration [29]. In this study, microcrystalline cellulose pellets were used to prepare compound essential oil pellets, which had a high drug loading rate and easy drug adhesion [30]. HPMC can be used as a binder, and the addition of HPMC can prolong the adhesion time of the drug so that the drug can better adhere to the pellets [31]. HPMC can be used as an enteric coating material for oral preparations to endow them with certain enteric properties [32]. This study investigated the pharmacokinetics of compound essential oil pellets in a pig model to provide support for future clinical administration. For the first time, GC‒MS/MS was used to simultaneously determine the contents of trans-anethole and cinnamaldehyde in pig plasma after oral administration of compound essential oil pellets, and the pharmacokinetic parameters of these drugs were studied. Generally, whether a drug has linear kinetics depends on whether the pharmacokinetic parameters AUC and C_max_ are directly correlated with the dosage [33]. In this study, it was found that the C_max_ and AUC of cinnamaldehyde and trans-anisole in compound essential oil pellets were directly correlated with the dosage, thus exhibiting linear kinetics. T_max_, MRT_0−∞_, and T_1/2_ were similar, and their values were independent of the dose.

## Conclusions

In summary, cinnamon oil and star anise oil showed good antibacterial effects against *E. coli* and *Salmonella*, and the combined use of the two drugs resulted in synergistic and additive effects. Compound essential oil pellets of cinnamon and star anise oils were successfully prepared by centrifugal granulation technology. The adhesive was HPMC, which provided the pellets with certain enteric properties. The good stability of the pellets was investigated by determining the content, particle size distribution, angle of repose, fragility, and stability of the pellets. An in vitro dissolution test showed that the composite essential oil pellets had intestinal properties. Pharmacokinetic studies showed that the compound essential oil pellets exhibited linear pharmacokinetic characteristics in a pig model.

### Electronic supplementary material

Below is the link to the electronic supplementary material.

Supplementary Material 1

## Data Availability

The datasets used and analyzed during the current study are available from the corresponding author upon reasonable request.

## Abbreviations

Tmax: time to reach maximum
Cmax: maximum concentration of drug
AUC0-∞: area under plasma concentration-time curve
Vd: volume of distribution
CL: clearance rate
MRT0-∞: mean resident time
T1/2: elimination half-life
F: bioavailability
*E. coli*: *Escherichia coli*
MIC: minimum inhibitory concentrations
PK: pharmacokinetics
API: active pharmaceutical ingredient
p.o.: oral administration
