# Supplementary material for: Characterization and pharmacokinetics of cinnamon and star anise compound essential oil pellets prepared via centrifugal granulation technology
Source: BMC Vet Res. 2024 May 9;20:184. doi: 10.1186/s12917-024-04026-7 (PMC11083769; doi:10.1186/s12917-024-04026-7)
Supplement: Supplementary file 1 — Supplementary Material 1 [file 12917_2024_4026_MOESM1_ESM.docx]

**Characterization and pharmacokinetics of cinnamon and star anise compound essential oil pellets prepared via centrifugal granulation technology Supplementary materials**

**Stability research**

**Accelerated stability test of compound essential oil pellets**

The stability of compound essential oil pellets was investigated through accelerated experiments. The results in table 1 showed that there was no significant change in the drug content of the compound essential oil pellets under accelerated stability conditions within 6 months.

Table 1 Results of accelerated stability test of compound essential oil pellets

| Batch | Time(mou) | Appearance | Content (Labeled quantity %) | |
| --- | --- | --- | --- | --- |
|  |  |  | Cinnamaldehyde | Trans-anethole |
| 1 | 0 | Pale yellow sphere | 2.48 | 1.29 |
|  | 1 | Pale yellow sphere | 2.49 | 1.29 |
|  | 2 | Pale yellow sphere | 2.48 | 1.28 |
|  | 3 | Pale yellow sphere | 2.42 | 1.21 |
|  | 6 | Pale yellow sphere | 2.38 | 1.18 |
| 2 | 0 | Pale yellow sphere | 1.96 | 1.17 |
|  | 1 | Pale yellow sphere | 1.96 | 1.17 |
|  | 2 | Pale yellow sphere | 1.95 | 1.16 |
|  | 3 | Pale yellow sphere | 1.90 | 1.11 |
|  | 6 | Pale yellow sphere | 1.87 | 1.06 |
| 3 | 0 | Pale yellow sphere | 2.36 | 1.32 |
|  | 1 | Pale yellow sphere | 2.35 | 1.32 |
|  | 2 | Pale yellow sphere | 2.34 | 1.31 |
|  | 3 | Pale yellow sphere | 2.21 | 1.21 |
|  | 6 | Pale yellow sphere | 2.18 | 1.18 |

**The long-term stability test results of compound essential oil pellets**

The stability of compound essential oil pellets was investigated through accelerated experiments. The results in table 2 showed that there was no significant change in the drug content of the compound essential oil pellets under long-term conditions within 6 months.

Table 2 Results of Long-term stability test results of compound essential oil pellets

| Batch | Time(mou) | Appearance | Content (Labeled quantity %) | |
| --- | --- | --- | --- | --- |
|  |  |  | Cinnamaldehyde | Trans-anethole |
| 1 | 0 | Pale yellow sphere | 2.48 | 1.29 |
|  | 3 | Pale yellow sphere | 2.49 | 1.29 |
|  | 6 | Pale yellow sphere | 2.48 | 1.28 |
|  | 9 | Pale yellow sphere | 2.47 | 1.27 |
|  | 12 | Pale yellow sphere | 2.35 | 1.19 |
| 2 | 0 | Pale yellow sphere | 1.96 | 1.17 |
|  | 3 | Pale yellow sphere | 1.96 | 1.17 |
|  | 6 | Pale yellow sphere | 1.96 | 1.17 |
|  | 9 | Pale yellow sphere | 1.95 | 1.16 |
|  | 12 | Pale yellow sphere | 1.82 | 1.05 |
| 3 | 0 | Pale yellow sphere | 2.36 | 1.32 |
|  | 3 | Pale yellow sphere | 2.36 | 1.31 |
|  | 6 | Pale yellow sphere | 2.35 | 1.31 |
|  | 9 | Pale yellow sphere | 2.34 | 1.30 |
|  | 12 | Pale yellow sphere | 2.21 | 1.21 |

**The** **high temperature and high light of compound essential oil pellets**

The stability of compound essential oil pellets was investigated through high temperature and high light experiments. The results in table 3 showed that there was no significant change in the drug content of the compound essential oil pellets under high temperature and high light conditions within 5 days.

Table3 The influencting factors of the compound essential oil pellets

| Influence Factors | | | Time（d） | | |
| --- | --- | --- | --- | --- | --- |
|  |  |  | 0 | 5 | 10 |
| High temperature | Appearance | | Pale yellow sphere | Pale yellow sphere | Pale yellow sphere |
|  | Content (Labeled quantity %) | Cinnamaldehyde | 2.48 | 2.4 | 2.35 |
|  |  | Trans-anethole | 1.29 | 1.21 | 1.18 |
| High light | Appearance | | Pale yellow sphere | Pale yellow sphere | Pale yellow sphere |
|  | Content (Labeled quantity %) | Cinnamaldehyde | 2.48 | 2.39 | 2.36 |
|  |  | Trans-anethole | 1.29 | 1.22 | 1.20 |
